# An Ultra-Short Baseline Positioning Model Based on Rotating Array & Reusing Elements and Its Error Analysis

**DOI:** 10.3390/s19204373

**Published:** 2019-10-10

**Authors:** Jinwu Tong, Xiaosu Xu, Lanhua Hou, Yao Li, Jian Wang, Liang Zhang

**Affiliations:** Key Laboratory of Micro-Inertial Instruments and Advanced Navigation Technology, Ministry of Education, School of Instrument Science and Engineering, Southeast University, Nanjing 210096, China; tongjinwu@seu.edu.cn (J.T.); lanhua_hou@seu.edu.cn (L.H.); liyao@seu.edu.cn (Y.L.); 230169708@seu.edu.cn (J.W.); zhangliang418@seu.edu.cn (L.Z.)

**Keywords:** navigation, SINS, USBL, high-precision, positioning calculation, rotating array, reusing elements, array elements spacing error elimination

## Abstract

The USBL (Ultra-Short Base Line) positioning system is widely used in underwater acoustic positioning systems due to its small size and ease of use. The traditional USBL positioning system is based on ‘slant range and azimuth’. The positioning error is an increasing function with the increase in distance and the positioning accuracy depends on the ranging accuracy of the underwater target. This method is not suitable for long-distance underwater positioning operations. This paper proposes a USBL positioning calculation model based on depth information for ‘rotating array and reusing elements’. This method does not need to measure the distance between the USBL acoustic array and target, so it can completely eliminate the influence of long-distance ranging errors in USBL positioning. The theoretical analysis and simulation experiments show that the new USBL positioning model based on ‘rotating array and reusing elements’ can completely eliminate the influence of the wavelength error and spacing error of underwater acoustic signals on the positioning accuracy of USBL. The positioning accuracy can be improved by approximately 90%, and the horizontal positioning error within a positioning distance of 1000 m is less than 1.2 m. The positioning method has high precision performance in the long distance, and provides a new idea for the engineering design of a USBL underwater positioning system.

## 1. Introduction

Due to the severe attenuation of electromagnetic waves under water, the application of high precision satellite navigation systems underwater is limited. As the positioning error of the INS (Inertial Navigation System) accumulates with time, one single INS cannot complete the high precision navigation and positioning task in the long-range alone [1]. Acoustic waves are the only information carriers that have been proven to be able to travel long distances under water [2]. Underwater acoustic positioning is the main means of underwater target positioning and tracking at present [3]. Pan-Mook Lee and others proposed the range aiding method to integrate with IMU(Inertial Measurement Unit) /DVL (Doppler Velocity Log) [4]. According to the length of the baseline, an underwater acoustic positioning system can be divided into LBL (Long Base Line), SBL (Short Base Line), and USBL, where USBL is a variant of SBL [5]. By virtue of the small size and convenient use, USBL is widely used in underwater positioning. At present, there are mature USBL products from Kongsberg in Norway; IXBlue in France; LinkQuest in the United States; and AAE, Nautronix, and Sonardyne in the UK. Among them, Kongsberg is one of the earliest research institutes for USBL acoustic positioning systems. Kongsberg launched the first generation USBL in 1996 and their new HiPAP700 is one of a few long-range USBL positioning systems where its working water depths can reach 10,000 m. However, a single SINS (Strap-down Inertial Navigation System) or USBL cannot complete underwater positioning tasks with high precision performance in long distance submerged travel. The SINS/USBL integrated navigation mode is widely adopted in existing underwater positioning products [6]. GAPS-USBL, as a high precision USBL positioning system launched by Ixblue in France, adopts a combined design of USBL/INS /GNSS (Global Navigation Satellite System). This integrated structure eliminates the installation error calibration between a transceiver array of USBL equipment and INS devices before use. Therefore, the influence of installation error angle on the positioning accuracy of USBL is reduced [7]. In [8,9,10,11], the loose combination and tight combination were adopted in the SINS/USBL integrated navigation positioning algorithm. Bingbing Gao and other researchers from Northwestern Polytechnical University proposed the SINS/USBL/DVL integrated navigation scheme [12]. Jianhua Cheng, Ping Dong and others from Harbin Engineering University proposed an INS/USBL nonlinear combination method based on relative measurement information for nonlinear problems [13]. The filtering theory of SINS/USBL integrated navigation is based on the mature filtering algorithms of SINS and other observation information such as SINS/GNSS [14] and SINS/DVL [15]. Since there is an installation error angle between the base surface of the acoustic array and the mounting base surface. The calibration is indispensable before used. Professor Hsin-Hung Chen from the National Sun Yat-Sen University of Taiwan proposed a multi-circle sailing and straight-line sailing method, which was verified experimentally that to be feasible [16,17]. A USBL installation error angle calibration method based on double transponders was proposed in 2018 [2], but the calibration process was more complicated. In 2019, a USBL installation error angle calibration single transponder USBL based on dual vector reconstruction was proposed, where high-precision and a fast calibration of the USBL installation error angle can be achieved [18]. 

The traditional USBL system adopts the narrow-band underwater acoustic signal design, which uses the phase difference or phase ratio method for the positioning calculation. In general, the larger the spacing of acoustic array elements, the higher the positioning accuracy of the USBL. Unfortunately, the spacing of the acoustic array elements cannot be designed to be infinite for two reasons. The main reason is that the large-scale array structure of the USBL loses its compaction, which is inconvenient to use. The other is that when the spacing of the acoustic array elements is greater than half a wavelength, the phase difference ambiguity problem will occur, which is a very troublesome problem. On this issue, Min Yu, a scholar from Wuhan University of Technology, proposed a method of expanding the spacing of array elements by adding auxiliary elements in her PhD thesis (Harbin Engineering University, China, 2006) [19]. This method not only improves the positioning accuracy of USBL, but also solves the problem of phase difference ambiguity. However, this method creates new problems that with the increase of the number of acoustic array elements, a large amount of work on the USBL element spacing error calibration is needed. In addition, as the number of array elements increases, the complexity of the manufacturing of USBL acoustic array devices increases, which will reduce the reliability of the USBL positioning system [20]. Therefore, a method to improve the accuracy of the USBL positioning calculation has been a research hotspot in this field. From the existing reference, the main method to improve the accuracy of the USBL positioning solution is to calibrate the installation error angle [16,21,22] and the spacing error between the acoustic array elements [23]. The existing technology can reduce the spacing error of the USBL acoustic array elements to less than 1 mm or even less than 0.12 mm [23], but the calibration process is extremely complex. With the increase of the usage time, the spacing of the elements will be changed. In particular, after replacing the acoustic array elements, this parameter needs to be corrected again. Yunhui Wang, a graduate student of the research team of Guiqing Sun from the Oceanography School of Zhejiang University proposed a USBL positioning model based on depth information [20]. In the long-distance situation, the model has better positioning accuracy than the traditional method base on slant range and the azimuth, and it is not needed to measure the distance, which improves the positioning efficiency, so it is especially suitable for the passive positioning of cooperative targets. However, the spacing error between different acoustic array elements is neglected, so this method fails to eliminate the influence of the element spacing error on the accuracy of USBL positioning. How to eliminate the influence of the spacing error array of elements in USBL positioning calculation is of great significance to improve the accuracy of the USBL positioning system.

To overcome the shortcoming where the positioning error is correlated with the positioning distance in the traditional USBL positioning algorithm, a new model for USBL positioning calculation has been designed. The passive positioning of the cooperative target is considered as the positioning object of this model researched in this paper. That is, there is no acoustic transmitter in the USBL acoustic array. The USBL positioning system does not emit underwater acoustic signals. The USBL positioning calculation depends on the depth information of the target and the phase difference between the acoustic array elements. This model can perform a USBL positioning calculation without slant distance measurement and can completely eliminate the influence of the spacing error on positioning accuracy, thus achieving a high-precision USBL positioning calculation.

The rest of this paper is structured as follows. Section 2 illustrates the basic working principle of the traditional USBL positioning system and the characteristics of the spacing error of the acoustic array elements, then analyses the horizontal positioning error of two positioning models based on the slant range and azimuth and phase difference ratio. Section 3 gives the design and error analysis of a USBL positioning model based on the rotating array and array element reusing in detail. This is also the main contribution of this paper. Section 4 presents the simulation and verification, and the numerical simulations of the proposed model are carried out and compared with the traditional positioning model. Section 5 is the conclusion and outlook.

## 2. The Principle and Error Analysis of Traditional USBL Positioning System Based on Distance and Angle of Incidence

### 2.1. Coordinate Frame Definition

**Figure 1:**
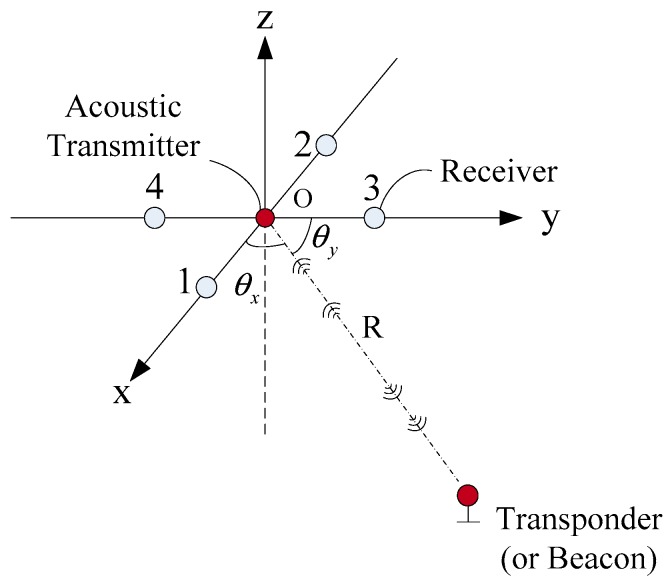
The schematic diagram of the Ultra-Short Base Line array structure based on four receiving array elements.

Multiple frames were involved in this paper: a USBL array frame $O_{U}$-$X_{U}Y_{U}Z_{U}$ (abbreviated as U-frame) with the Z axis pointing upward; the three coordinate axes were set in the right-hand frame, and the coordinate axes were orthogonal to each other. Receiving array elements 1, 2, 3, and 4 were located on the *x*-axis and *y*-axis, respectively, as shown in Figure 1. It also can be seen that the acoustic emitter was located at the origin of the U-frame, which is the acoustic center of the acoustic array (there is no acoustic emitter in the USBL acoustic array with passive location mode). The USBL position calculation of the target was carried out in the U-frame.

**Figure 2:**
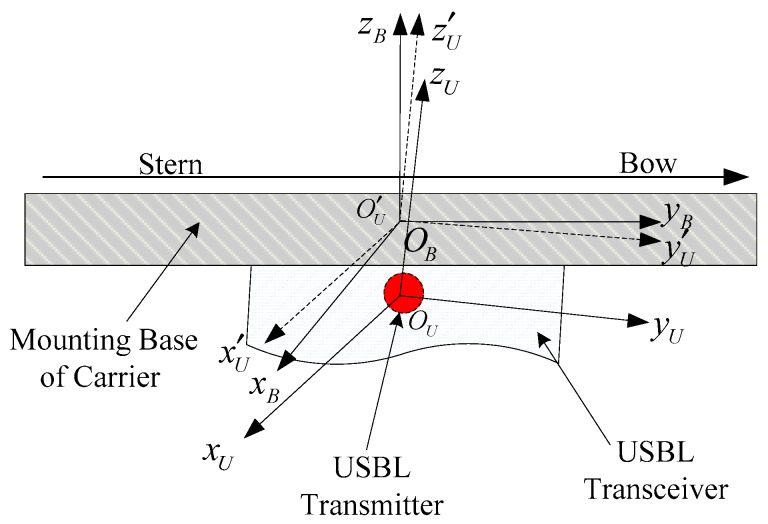
The diagram of the receiving and sending array with the angle misalignment error.

The body frame (or base frame $O_{B}-X_{B}Y_{B}Z_{B}$, abbreviated as the B-frame) refers to the frame of the carrier where the USBL transceiver array equipment is installed. The positive direction of the y-axis points to the front of the bow line. The *x*-axis points to the starboard side. The *z*-axis points upward, which is perpendicular to the plane xoz. Ideally, the B-frame coincides with the origin of the U-frame, and the axes are also completely parallel. However, in engineering application, the origins of the frames are not completely coincident, and there is an angular deviation between their corresponding axes, that is, the angle misalignment error of the USBL, which is shown as Figure 2.

In the global frame ($O_{G}-X_{G}Y_{G}Z_{G}$, abbreviated as the G-frame), the x-axis points to the east, the y-axis points to the north, and the z-axis points upward. The WGS-84 frame, where the absolute position of the target can be obtained, was adopted in this paper.

### 2.2. The Basic Principle of the USBL Positioning System

It can be seen from Figure 1 that the basic equations of the USBL positioning algorithm based on the slant range and azimuth are as follows.

(1) $$x_{u}=R\cos\theta_{x}$$

(2) $$y_{u}=R\cos\theta_{y}$$

(3) $$\begin{array}{ll} z_{u} & =-\sqrt{R^{2}-X_{u}{}^{2}-Y_{u}{}^{2}}\\ & =-R\sqrt{1-\cos^{2}\theta_{x}-\cos^{2}\theta_{y}} \end{array}$$

(4) R=CT

where $x_{u}$, $y_{u}$, and $z_{u}$ are the calculated values of the USBL positioning in the U-frame. $\theta_{x}$ is the angle between the slant range and the x-axis. $\theta_{y}$ is the angle between the slant range and the y-axis. $\theta_{z}$ is the angle between the slant range and the z-axis. R is the distance between the center of the acoustic array and target. C is the equivalent underwater acoustic velocity and T is the one-way propagation time of the acoustic signal.

**Figure 3:**
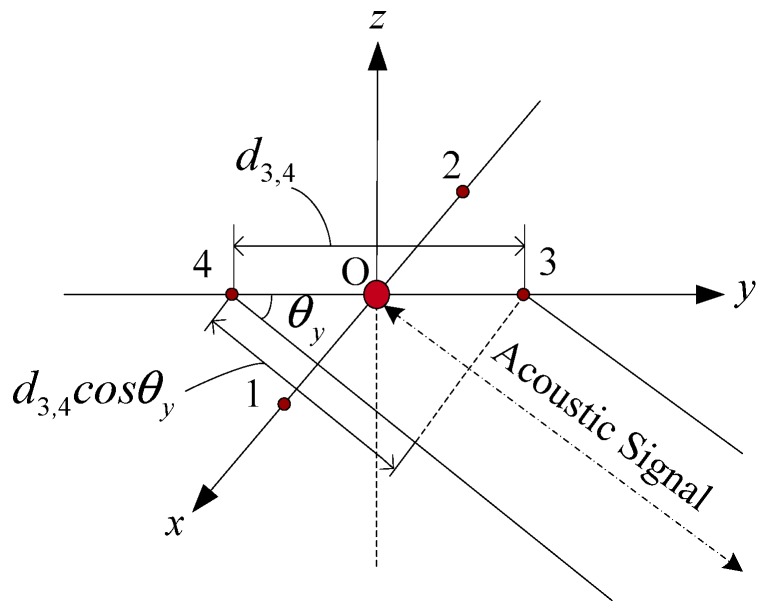
Diagram of the USBL phase difference.

Figure 3 is the diagram of the USBL phase difference.

(5) $$\varphi =\frac{2\pi d}{\lambda }\cos\theta$$

(6) λ=C/f

Equation (5) represents the phase difference between the two array elements, where d is the distance between the two array elements; θ is the direction angle of the acoustic signal; λ is the wavelength of the underwater acoustic signal; f is the frequency of the underwater acoustic signal; and C is the underwater acoustic velocity. From Equation (5), it can be obtained that

(7) $$\theta_{x}=\arccos\left(\frac{\lambda \varphi_{1,2}}{2\pi d_{1,2}}\right)$$

(8) $$\theta_{y}=\arccos\left(\frac{\lambda \varphi_{3,4}}{2\pi d_{3,4}}\right)$$

Substituting Equations (7) and (8) into Equations (1) and (2)

(9) $$x_{u}=\frac{R\lambda \varphi_{1,2}}{2\pi d_{1,2}}$$

(10) $$y_{u}=\frac{R\lambda \varphi_{3,4}}{2\pi d_{3,4}}$$

where λ is the wavelength; $\varphi_{1,2}$, $\varphi_{3,4}$ are the phase difference of 1–2, and 3–4, respectively; and $d_{1,2}$, $d_{3,4}$ are the spacing of array elements 1–2 and 3–4, respectively. Equations (9), (10), and (3) are the basic formulas of the USBL positioning system.

### 2.3. Array Element Spacing Error Modeling

The array element spacing error is one of the main error sources of the USBL positioning calculation. The high-precision calibration and compensation for the element spacing error can effectively suppress the USBL positioning error. High-precision calibration methods can be used in most underwater positioning applications; however, under the existing technical conditions, due to the limited calibration accuracy of the spacing error, the existing calibration methods in high-precision and ultra-high-precision underwater applications still cannot meet the requirements of positioning accuracy. How to suppress or completely eliminate the influence of elements spacing error on USBL positioning accuracy is still a hot topic in this field. This section establishes a USBL array elements spacing error model to study the characteristics of the USBL array elements spacing error.

**Figure 4:**
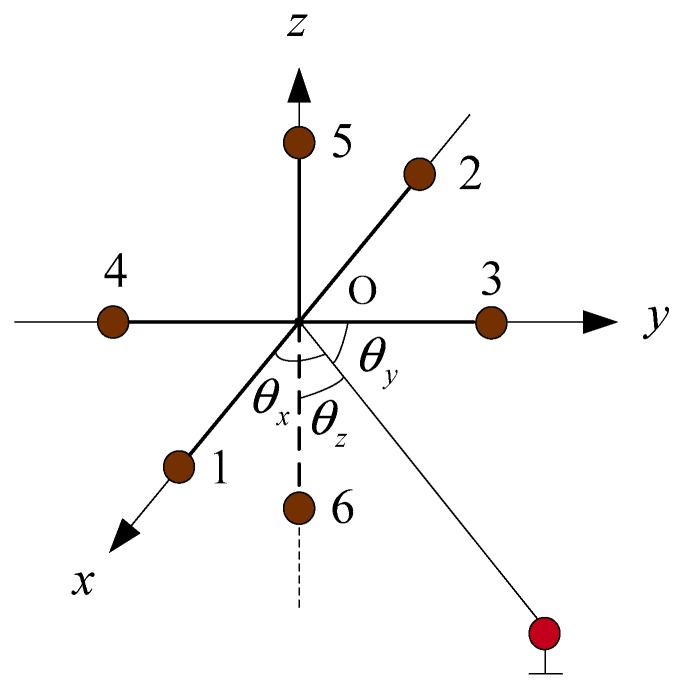
The schematic diagram of the USBL array structure based on six receiving array elements.

The USBL array elements spacing error can be divided into the measurement error $\delta d_{m}$ and the device drift error $\delta d_{d}$. The measurement error $\delta d_{m}$ is the main error, which has a direct impact on the accuracy of the USBL positioning calculation. The error is constant in one measurement. Device drift error $\delta d_{d}$ refers to the spacing error caused by the acoustic center drift of the element and the change in device geometry caused by rust and aging, which is very small and has little change over a long time. It usually has little influence on the positioning accuracy in engineering applications and therefore was neglected in this paper.

(11) $$\tilde{d}=d+\delta d$$

(12) $$\delta d=\delta d_{m}+\delta d_{d}$$

(13) $$\delta {\dot{d}}_{m}=0$$

where $\tilde{d}$ is the measured value of the element spacing; d is the truth-value of the element spacing; δd is the error of the element spacing; $\delta d_{m}$ is the measurement error which is the constant error; and $\delta d_{d}$ is the drift error. Figure 4 shows the schematic diagram of the USBL receiving array based on six receiving array elements. 

The array elements 1, 2, 3, 4, 5, and 6 are all located on the coordinate axis. The intersection point of the connection of array elements 1–2, 3–4, and 5–6 is the coordinate origin. The spacing of array elements 1–2, 3–4, and 5–6 are as follows:

(14) $${\tilde{d}}_{1,2}=d_{1,2}+\delta d_{1,2}$$

(15) $${\tilde{d}}_{3,4}=d_{3,4}+\delta d_{3,4}$$

(16) $${\tilde{d}}_{5,6}=d_{5,6}+\delta d_{5,6}$$

where ${\tilde{d}}_{i,j}$ is the measurement values of the spacing between array elements i and j; $d_{i,j}$ is the true value of the spacing between array elements i and j; $\delta_{i,j}$ is the spacing error between array elements i and j.

### 2.4. The Error Analysis of the USBL Positioning Solution Based on the Slant Range and Azimuth Method

Using the complete differential method in Equation (9), the positioning error equation of the USBL positioning solution in the x-axis can be obtained.

(17) $$\delta x_{u}=\frac{R\lambda }{2\pi d_{1,2}}\delta \varphi_{1,2}+\frac{R\varphi_{1,2}}{2\pi d_{1,2}}\delta \lambda +\frac{\lambda \varphi_{1,2}}{2\pi d_{1,2}}\delta R-\frac{R\lambda \varphi_{1,2}}{2\pi }\frac{\delta d_{1,2}}{d_{1,2}{}^{2}}$$

Considering that each error item is independent of each other, the mean square error of the target location solution value in the x-axis direction is as follows:

(18) $$\delta d_{x}^{2}={\left(\frac{R\lambda \varphi_{1,2}}{2\pi d_{1,2}}\right)}^{2}({\left(\frac{\delta \varphi_{1,2}}{\varphi_{1,2}}\right)}^{2}+{\left(\frac{\delta \lambda }{\lambda }\right)}^{2}+{\left(\frac{\delta R}{R}\right)}^{2}+{\left(\frac{\delta d_{1,2}}{d_{1,2}{}^{}}\right)}^{2})$$

where $\delta \varphi_{1,2}$ is the phase difference error; δλ is the wavelength error; and δR is the ranging error and $\delta d_{1,2}$ is the spacing error between the array elements 1 and 2. δR/R is the relative distance error factor; δλ/λ is the relative wavelength error factor; and $\delta d_{1,2}/d_{1,2}$ is the relative spacing error factor. 

In order to analyze the influence of errors, Equation (17) is rewritten in the form of relative errors.

(19) $$\frac{\delta x_{u}}{R}=\frac{\lambda \varphi_{1,2}}{2\pi d_{1,2}}(\frac{\delta R}{R}+\frac{\delta \lambda }{\lambda }-\frac{\delta d_{1,2}}{d_{1,2}})+\frac{\lambda \delta \varphi_{1,2}}{2\pi d_{1,2}}$$

According to Equation (5) rewriting Equation (19), it can be obtained that

(20) $$\frac{\delta x_{u}}{R}=\cos\theta_{x}(\frac{\delta R}{R}+\frac{\delta \lambda }{\lambda }-\frac{\delta d_{1,2}}{d_{1,2}})+\frac{\lambda \delta \varphi_{1,2}}{2\pi d_{1,2}}$$

With Equations (4) and (6), Equation (20) can be written as follows:

(21) $$\frac{\delta x_{u}}{R}=\cos\theta_{x}(\frac{\delta T}{T}+2\frac{\delta C}{C}-\frac{\delta d_{1,2}}{d_{1,2}})+{\frac{\lambda \delta \varphi_{1,2}}{2\pi d_{1,2}}}^{}$$

where δT is the time delay measurement error; δC is the equivalent acoustic velocity estimation error; δT/T is the relative time delay measurement error factor; and δC/C is the relative equivalent acoustic velocity estimation error factor. From Equation (20), it can be seen that the USBL positioning error is caused by the time delay measurement error δT, the equivalent acoustic velocity estimation error δC, and the spacing error of array elements $\delta d_{1,2}$ is related to the incident angle $\theta_{x}$. When the target is located right below the acoustic array, that is, the value of the incidence angle $\theta_{x}$ is close to 90°, the positioning error of the USBL is less affected by these three errors and is mainly affected by the phase difference error. Therefore, the positioning accuracy of the USBL has the best performance in the range of cone angle directly below it.

When the target is not directly under the USBL transceiver array, in addition to the phase difference error δφ, the USBL positioning error caused by the time delay measurement error δT, the equivalent acoustic velocity error δC, and the spacing error of the array elements δd should not be neglected. Based on the performance parameters of the current products, the effects of these three errors were analyzed and the details are shown in Table 1.

Considering the location environment with a location distance of 1000 m, an equivalent underwater acoustic velocity of 1500 m/s, and a distance of the USBL array elements of 250 mm, which is shown in Table 2. 

The relative time delay measurement error factor is as follows:

(22) $$\frac{\delta T}{T}\approx 0.0015$$

where δT is the time delay error term, and its relative error decreases with the increase in the positioning distance without considering the acoustic line bending. The relative equivalent acoustic velocity estimation error factor is

(23) $$2\frac{\delta C}{C}=\;0.0000666$$

The relative spacing error factor is

(24) $$\frac{\delta d}{d_{1,2}}=0.008$$

The equivalent acoustic velocity estimation error term and the spacing error term are relatively stable values. Comparing Equations (22)–(24), it can be seen that the relative spacing error factor was much larger than the relative time delay measurement error factor and the relative equivalent acoustic velocity estimation error factor. Therefore, it is indispensable to calibrate the spacing error of the array elements δd prior to using it in a high-precision underwater acoustic positioning environment.

### 2.5. The Error Analysis of USBL Positioning Calculation Based on the Phase Ratio Method

In Figure 4, taking the array elements spacing error into account, the phase differences between two array elements on the same coordinate axis are as follows:

(25) $$\varphi_{1,2}=\frac{2\pi {\tilde{d}}_{1,2}\cos\theta_{x}}{\lambda }$$

(26) $$\varphi_{3,4}=\frac{2\pi {\tilde{d}}_{3,4}\cos\theta_{y}}{\lambda }$$

(27) $$\varphi_{5,6}=\frac{2\pi {\tilde{d}}_{5,6}\cos\theta_{z}}{\lambda }$$

According to the calculation method of the USBL based on the slant range and azimuth, it can be obtained that

(28) $$\cos\theta_{x}=\frac{x}{R}$$

(29) $$\cos\theta_{z}=\frac{z}{R}=\frac{h}{R}$$

where h is the depth of the target. From Equations (25), (27), and (28), it can be derived that

(30) $$\tilde{x}=R\cdot \cos\theta_{x}=\frac{h}{\cos\theta_{z}}\cdot \cos\theta_{x}=h\cdot \frac{\varphi_{1,2}}{\varphi_{5,6}}\cdot \frac{{\tilde{d}}_{5,6}}{{\tilde{d}}_{1,2}}$$

(31) $$\tilde{x}=x\cdot \frac{{\tilde{d}}_{5,6}}{{\tilde{d}}_{1,2}}$$

where $\tilde{x}$ is the coordinate value of the target in the U-frame, only considering the influence of the element spacing error on the USBL positioning accuracy and x is the coordinate value of the target in the USBL U-frame without considering each error term. In [20], the array elements spacing error was ignored. In fact, not only is there a spacing error between the elements, but the spacing error between the different elements is also different, that is $d_{1,2}\neq d_{3,4}\neq d_{5,6}$. From Equations (14) and (16), it can be known that

(32) $$\frac{{\tilde{d}}_{5,6}}{{\tilde{d}}_{1,2}}=\frac{d+\delta d_{5,6}}{d+\delta d_{1,2}}=\frac{d}{d+\delta d_{1,2}}+\frac{\delta d_{5,6}}{d+\delta d_{1,2}}\approx 1+\frac{\delta d_{5,6}}{d+\delta d_{1,2}}$$

Substituting Equation (32) into Equation (31), it can be obtained that

(33) $$\tilde{x}\approx x\cdot \left(1+\frac{\delta d_{5,6}}{d+\delta d_{1,2}}\right)$$

It can be seen from Equation (33) that there are two effective methods to improve the horizontal positioning accuracy, reduce the element spacing error $\delta d_{5,6}$, and expand the element spacing δd. Numerical simulations based on these two methods are performed below. 

### 2.6. The Simulation and Analysis of the Spacing Error of Array Elements 

In order to study the influence of the spacing error of array elements on the horizontal positioning accuracy, the influence of the spacing error of array elements on the horizontal positioning accuracy in the horizontal positioning range of 4000 m was studied, regardless of other error terms. Research was conducted on the horizontal positioning error (x-axis direction) caused by the spacing error of array elements of 250 mm with errors of 2 mm and 1 mm. The calibration method studied in [23] could reduce the spacing error of array elements less than 0.12 mm after compensation. This paper also used the numerical simulations and analyses of the spacing error of array elements of 12 mm.

It can be seen from Table 3. that by using the current ideal element calibration method [23] in a positioning environment where the horizontal distance is 4000 m and the element spacing error is less than 0.12 mm, the horizontal positioning error caused by the elements spacing error was 1.9191 m, a small positioning error that is hard to achieve in mainstream products. With a distance of 1000 m, the 1 mm elements spacing error could bring about a 4 m horizontal positioning error, and the 2 mm element spacing error could bring about a 7.9 m horizontal positioning error. This is an intolerable error term in high precision underwater positioning environments. For example, in order to reduce the positioning accuracy to 2 m with the slanting distance of 1000 m, the depth of 100 m, and the horizontal distance of about 995 m by only considering the elements spacing error, the element spacing error should be less than 0.5 mm. However, when considering the influence of other error items in the engineering project, the elements spacing error value should be smaller to meet the design requirements.

It is mentioned in the literature that increasing the spacing of the array elements can suppress the influence of the array elements spacing error on the positioning of the USBL. In the case of d = 500 mm, a simulation was also carried out. The details are shown as Table 4. 

It can be seen from Table 3 and Table 4 that when the spacing of the array elements is doubled, the USBL horizontal positioning error term caused by the equivalent element spacing error was about half of the original. Therefore, increasing the spacing of the array elements can indeed suppress the USBL horizontal positioning error caused by the spacing error of the array elements. However, the consequence of the large spacing of the array elements is that the size of the USBL array becomes larger, so the portability of the USBL will be affected. In addition, it also brings about a phase difference ambiguity problem that needs to be solved. Furthermore, as the spacing of the array elements increases, the spacing error of the array elements will also increase. Therefore, increasing the spacing of the array elements is not an ideal means by which to effectively suppress the influence of the spacing error of array elements on the USBL positioning.

## 3. A USBL Positioning Model and Its Error Analysis Based on Rotating Array and Reusing Elements

### 3.1. The Design of the Algorithmic Model 

The method based on phase ratio in [20] neglected the influence of the spacing error of the array element. In this paper, we considered that there was a spacing error between the array elements, and that there were differences in the spacing errors between different groups of array elements, as seen in Figure 4.

(34) $$\delta d_{1,2}\neq \delta d_{3,4}\neq \delta d_{5,6}$$

Since the truth-value of the spacing of array elements $d_{i,j}$ is unknown, and the spacing error of array elements $\delta d_{i,j}$ is also difficult to calibrate accurately, this paper designed a USBL positioning calculation model based on the rotating array and reusing elements method, which is used to eliminate the influence of the spacing error of array elements on the USBL positioning system.

According to Equations (14)–(16) and (34), the purpose of this model is to equalize the spacing errors between the three elements groups, that is,

(35) $$\delta d_{1,2}=\delta d_{3,4}=\delta d_{5,6}$$

**Figure 5:**
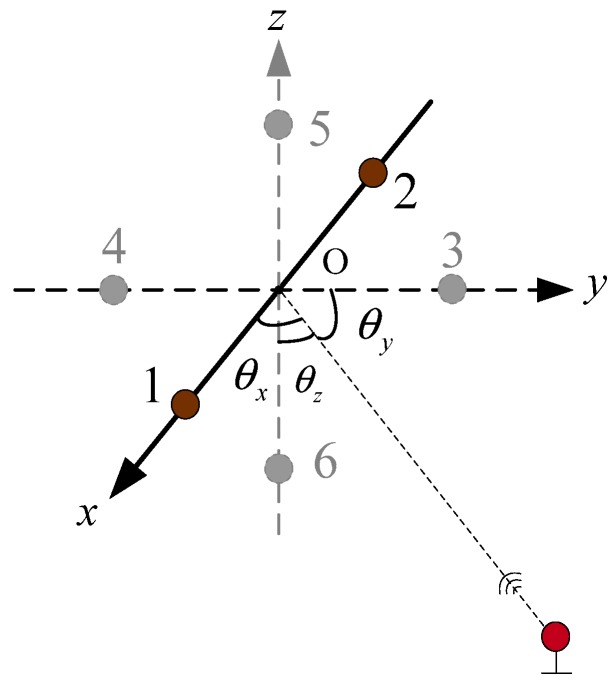
The schematic diagram of the USBL array structure based on the ‘rotating array and reusing elements’.

The spacing error between array elements is difficult to calibrate accurately. To make the three spacing errors completely equal, the most appropriate method is to use the elements reusing method. Thus, array elements 3, 4, 5, and 6 were removed, leaving only array elements 1 and 2, as shown in Figure 5. When it is necessary to calculate the phase difference $\varphi_{3,4}$ and the phase difference $\varphi_{5,6}$, the coordinate system is rotated so that array elements 1 and 2 on the coordinate axis are respectively rotated to the positions of elements 3–4 and 5–6, and the phase differences $\varphi_{3,4}$ and $\varphi_{5,6}$ are respectively calculated. In this process, only two array elements connected rigidly are used. In the whole positioning process, the spacing error between the two array elements is always $\delta d_{1,2}$.

Figure 5 shows the USBL structure based on virtual six receiving elements array. The array was composed of array elements 1 and 2, which were connected rigidly. The positioning principle of the USBL acoustic receiving array was the same as that of the 6-element USBL receiving array shown in Figure 4, and the error principle also was the same.

**Figure 6:**
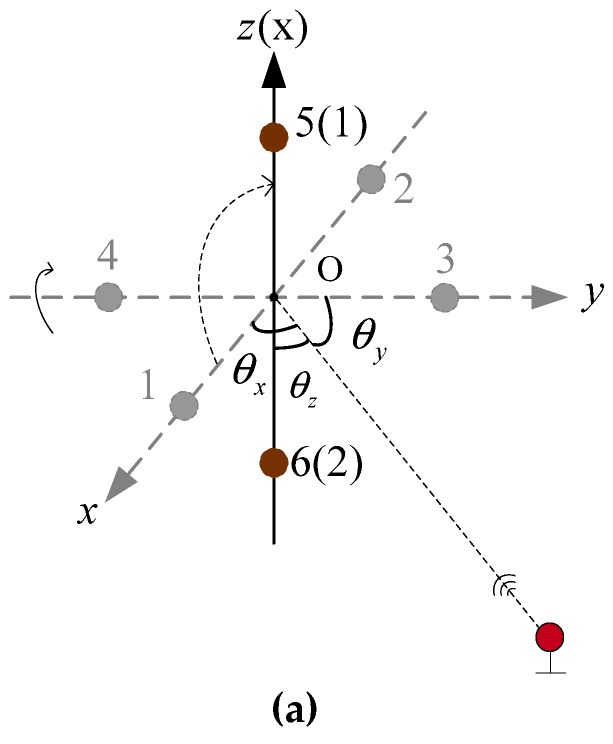

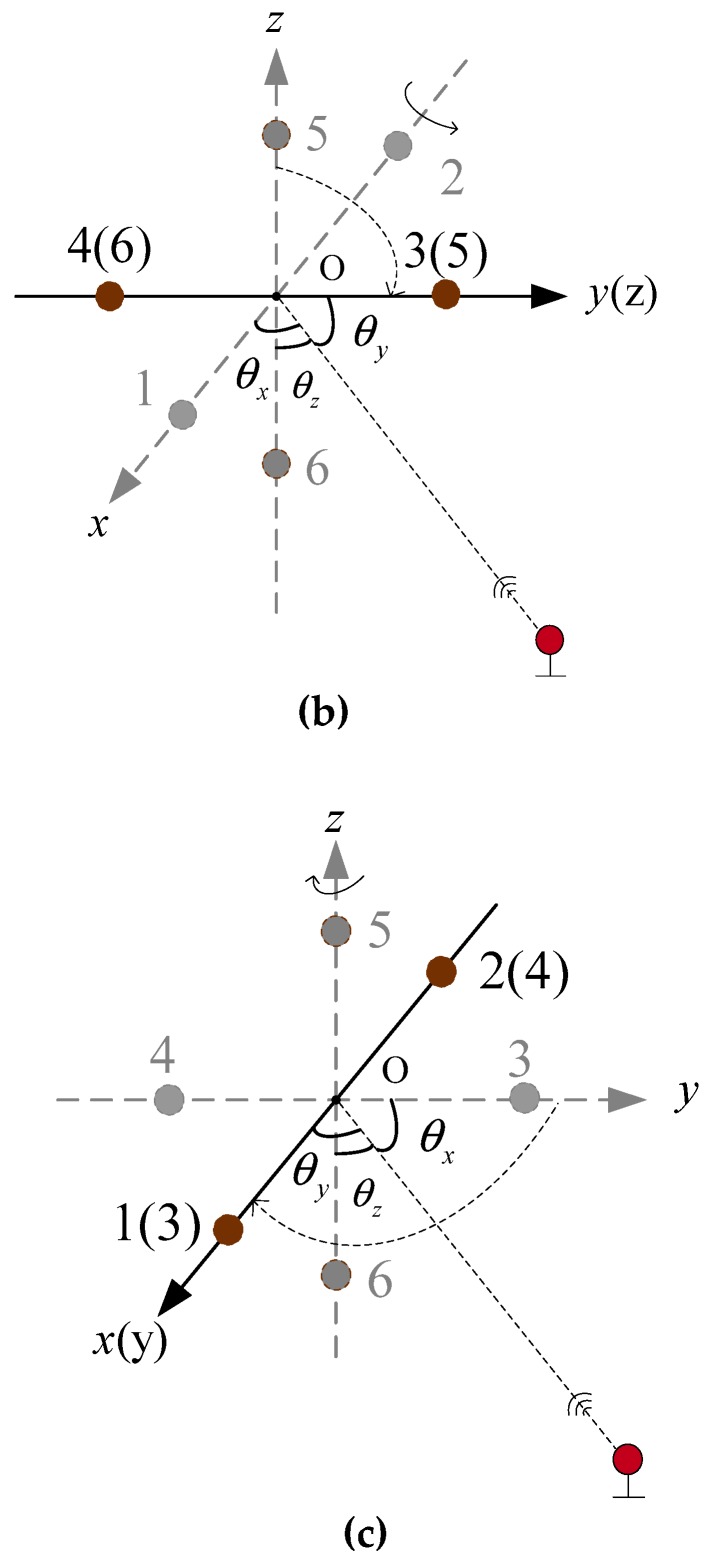
The rotating schematic diagram of the USBL array based on the rotating array and reusing elements. (**a**) The schematic diagram of the U-frame rotating 90° clockwise around the y-axis; (**b**) the schematic diagram of the U -frame rotating 90° clockwise around the x -axis; and (**c**) the schematic diagram of the U -frame rotating 90° clockwise around the z-axis.

Figure 6 shows the rotating schematic diagram of the USBL array based on the rotating array and reusing elements. Figure 6a is the schematic diagram of the U-frame rotating 90° clockwise around the *y*-axis, so that array elements 1 and 2 of the original coordinate *x*-axis can rotate to the position of array elements 5 and 6 on the *z*-axis. Similarly, Figure 6b is the schematic diagram of the U-frame rotating 90° clockwise around the x-axis, so that array elements 5 and 6 on the original *z*-axis can rotate to the position of array elements 3 and 4 on the *y*-axis. Figure 6c is the schematic diagram of the USBL array U-frame rotating 90° clockwise around the z-axis, so that array elements 3 and 4 on the original y-axis can rotate back to the position of the array elements 1 and 3 on the *x*-axis.

**Figure 7:**
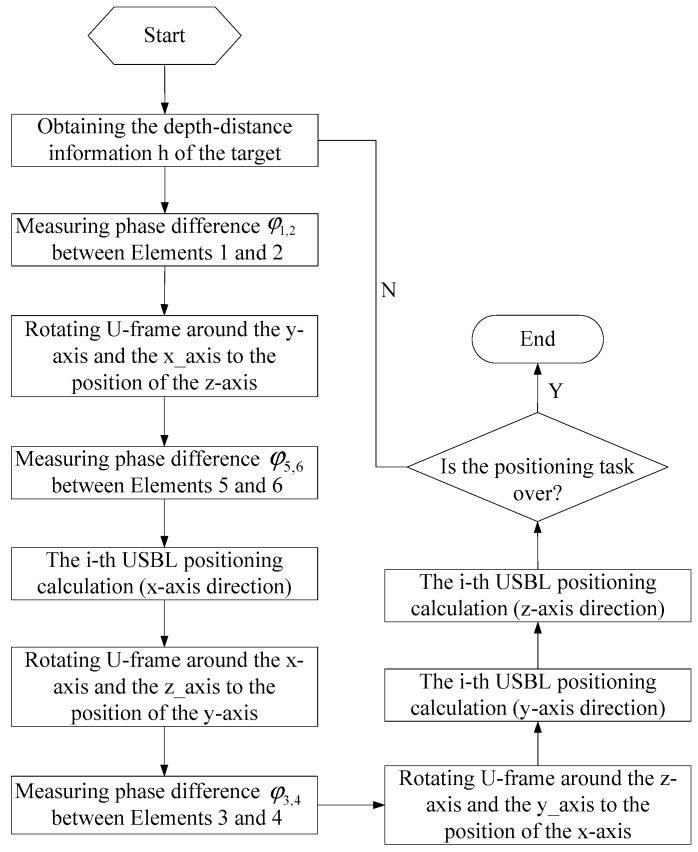
The flow chart of the USBL positioning calculation model based on the rotating array and reusing elements.

The calculation process is shown as follows:

Figure 7 depicts the flow chart of the USBL positioning calculation model based on the rotating array and reusing elements. First, the USBL positioning system acquires the depth information of the target $h_{t}$. Then, the USBL array obtains the phase difference $\varphi_{1,2}$ between array elements 1 and 2 by receiving the underwater acoustic signal from the target.

After acquiring the phase difference $\varphi_{1,2}$, the U-frame is rotated around the y-axis to rotate the x-axis to the position of the z-axis, and the phase difference $\varphi_{5,6}$ is measured. Similarly, the U-frame is rotated from the z-axis around the x-axis to the position of the y-axis, and the phase difference $\varphi_{3,4}$ is measured. After the three phase differences $\varphi_{1,2}$, $\varphi_{5,6}$, and $\varphi_{3,4}$ are collected, the i-th USBL horizontal positioning calculation is performed. Then, the U-frame is rotated around the z-axis and the y-axis is rotated back to the x-axis. At this time, if the positioning task is not finished, the program enters the next round of the target positioning solution, otherwise the positioning task is completed and ends this procedure.

In Figure 6a,

(36) $$\varphi_{1,2}=\frac{2\pi {\tilde{d}}_{1,2}\cos\theta_{x}}{\lambda }$$

where $\varphi_{1,2}$ is the phase difference between array elements 1 and 2; ${\tilde{d}}_{1,2}$ is the spacing value between array elements 1 and 2, which contains the constant spacing error $\delta d_{1,2}$; and $\theta_{x}$ is the angle between the underwater acoustic signal line and the x-axis.

In Figure 6b,

(37) $$\varphi_{5,6}=\frac{2\pi {\tilde{d}}_{5,6}\cos\theta_{z}}{\lambda }$$

where $\varphi_{5,6}$ is the phase difference between array elements 5 and 6; ${\tilde{d}}_{5,6}$ is the spacing value between array elements 5 and 6, which contains the constant spacing error $\delta d_{5,6}$; and $\theta_{z}$ is the angle between the underwater acoustic signal line and the z-axis.

It can be derived from Equation (30) that

(38) $$\tilde{x}=h\cdot \frac{\varphi_{1,2}}{\varphi_{5,6}}\cdot \frac{{\tilde{d}}_{5,6}}{{\tilde{d}}_{1,2}}$$

Since array elements 5 and 6 are obtained by rotating array elements 1 and 2 of the x-axis on the USBL acoustic array around the y-axis to the z-axis, and the two elements are rigidly connected before and after the rotation, it can be seen that ${\tilde{d}}_{1,2}={\tilde{d}}_{5,6}$. Thus, Equation (38) can be written as

(39) $$x=\tilde{x}=h\cdot \frac{\varphi_{1,2}}{\varphi_{5,6}}$$

**Figure 8:**
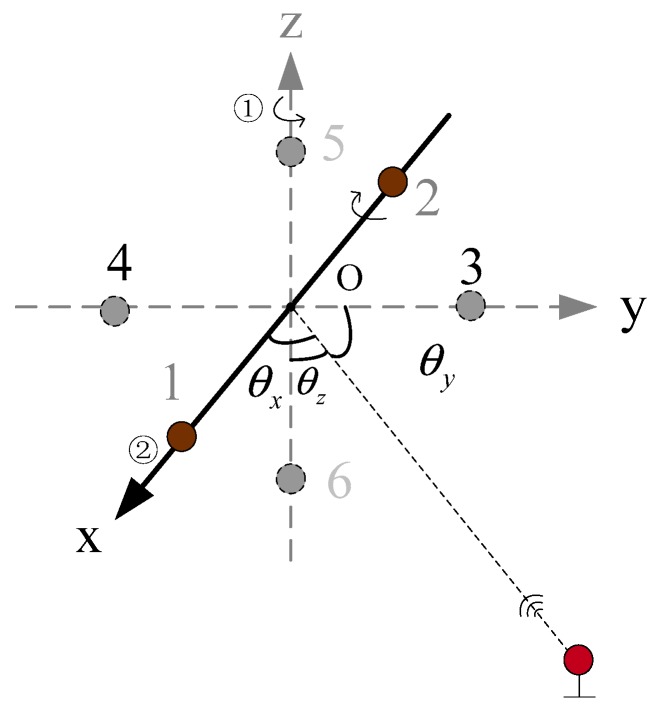
The rotation diagram of the USBL acoustic array.

The calculation of the *y*-axis coordinate value of the target T in the USBL U-frame is performed as follows. First, the USBL matrix U-frame is rotated around the z-axis, so that array elements 1 and 2 on the x-axis are rotated to the positions of array elements 3 and 4 on the y-axis, and the phase difference $\varphi_{3,4}$ is measured. Then, the U-frame is rotated around the x-axis, so that array elements 3 and 4 on the y-axis are rotated to the positions of array elements 5 and 6 on the z-axis, and the phase difference $\varphi_{5,6}$ is measured. Finally, the y-axis coordinate value of the target T in the U-frame is calculated according to the depth value h of the target T and the phase difference values $\varphi_{3,4}$ and $\varphi_{5,6}$. The USBL acoustic array structure and its rotation sequence are shown in Figure 8**.**

In order to save the positioning calculation time and adapt to the dynamic positioning environment, the horizontal position of the target *T* can be solved in the order shown in Figure 7. After the USBL acoustic positioning system completes the x-axis positioning calculation, the phase difference $\varphi_{3,4}$ of array elements 3 and 4 on the y-axis can be obtained immediately. As shown in Figure 6b, after acquiring the phase difference $\varphi_{5,6}$ of array elements 5 and 6 on the z-axis, the U-frame rotates around x-axis, and rotates array elements 5 and 6 on the z-axis to the position of elements 3 and 4 on the y-axis to measure the phase difference $\varphi_{3,4}$. Then, combined with the target depth value h, complete the positioning calculation of the target (y-axis direction) in the U-frame.

(40) $$y=R\cdot \cos\theta_{y}=\frac{h}{\cos\theta_{z}}\cdot \cos\theta_{y}=h\cdot \frac{\varphi_{3,4}}{\varphi_{5,6}}$$

(41) $$z=-\left|h_{T}-h_{A}\right|$$

where $h_{T}$ is the depth value of the target and $h_{A}$ is the depth value of the USBL acoustic array. Equations (39)–(41) are the basic equations for the USBL positioning calculation based on rotating array and reusing elements method. This positioning method is suitable for USBL positioning calculations where the target depth $h_{T}$ is known.

It can be seen from Equation (39) that the positioning accuracy of the USBL is only related to the depth value $h_{T}$ of the target and the error of the two phase differences $\varphi_{1,2}$ and $\varphi_{5,6}$. The positioning accuracy is independent of the spacing of the array elements $d_{i,j}$. Therefore, this model can completely eliminate the influence of the spacing error on the positioning accuracy of the USBL.

### 3.2. Error Analysis

In this section, the error source analysis of the USBL positioning model based on rotating array and reusing elements is performed. Apply the complete differential to Equation (39), and the positioning error (x-axis) of the target in the U-frame can be obtained.

(42) $$\begin{array}{ll} \delta x & =\delta h\cdot \frac{\varphi_{1,2}}{\varphi_{3,4}}+h\cdot \frac{\delta \varphi_{1,2}\cdot \varphi_{3,4}-\varphi_{1,2}\delta \varphi_{3,4}}{\varphi_{3,4}{}^{2}}\\ & =h\frac{\varphi_{1,2}}{\varphi_{3,4}}(\frac{\delta h}{h}+\frac{\delta \varphi_{1,2}}{\varphi_{3,4}}-\frac{\delta \varphi_{3,4}}{\varphi_{3,4}}) \end{array}$$

Under the condition that each error term is independent of each other, the mean square error of the target in the x-axis of the U-frame is

(43) $$\delta d_{x}^{2}={\left(h\frac{\varphi_{1,2}}{\varphi_{3,4}}\right)}^{2}({\left(\frac{\delta h}{h}\right)}^{2}+{\left(\frac{\delta \varphi_{1,2}}{\varphi_{3,4}}\right)}^{2}+{\left(\frac{\delta \varphi_{3,4}}{\varphi_{3,4}}\right)}^{2})$$

Contrasting Equation (43) and Equation (18), it can be found that when compared with the USBL positioning calculation method based on the slant range and azimuth method, the proposed USBL positioning calculation method based on the rotating array and reusing elements method eliminates the horizontal positioning error caused by the signal wavelength error δλ and the USBL element spacing error δd. The slant range measurement error δR is replaced by the depth measurement error δh. In the case of long distance, the depth measurement error is much smaller than the slant measurement error.

**Figure 9:**
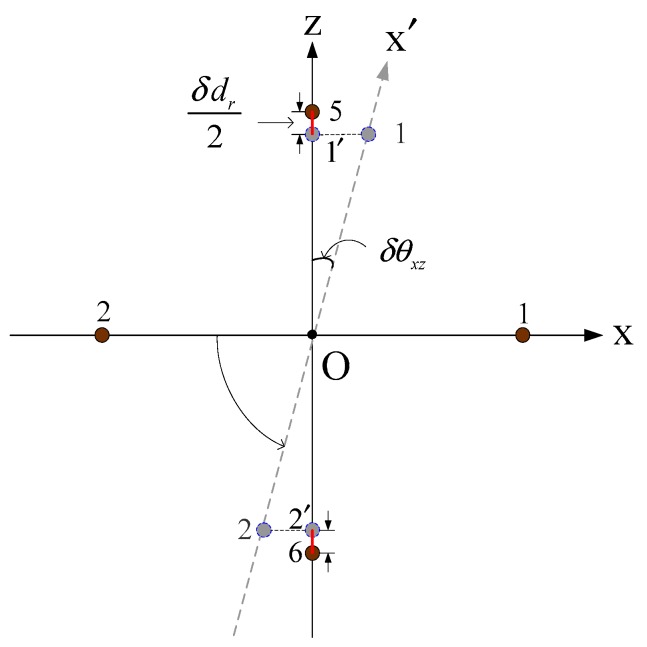
Schematic diagram of the USBL rotation error angle.

In the case of high-precision underwater positioning, the USBL positioning model based on the rotating and array element reuse should consider the USBL horizontal positioning error caused by the rotary angle error of the rotating device. During the process of the rotary device rotation, it is difficult to rotate the rotary device at exactly 90°. When the line connecting the two array elements after rotation does not completely coincide with the target coordinate axis, the distance between the two array elements projected onto the coordinate axis will be less than the spacing value of the actual array elements. As shown in Figure 6a, the acoustic array coordinate system U is rotated about the y-axis, rotating the array element 1–2 on the x-axis to the positions of the array elements 5–6 on the z-axis. However, in the actual rotation process, when the $x^{\prime }$-axis and the z-axis do not completely coincide, there is a small error angle $\delta \theta_{x^{\prime }z}$ between them, as shown in Figure 9, where the projection of the array elements 1–2 on the $x^{\prime }$-axis are array elements $1^{\prime }$–$2^{\prime }$. The distance between them is defined as the actual array spacing ${\tilde{d}}_{1,2}$.

The space error $\delta d_{r}$ of the array elements caused by the rotation error of the rotary device is

(44) $$\delta d_{r}=d_{5,6}-d_{1^{\prime },2^{\prime }}$$

In Figure 9

(45) $$\begin{array}{ll} \frac{\delta d_{r}}{2} & =d_{o,5}-d_{o,1^{\prime }}\\ & =d_{o,5}-d_{o,1}\times \cos\delta \theta_{xz}\\ & =\frac{d}{2}-\frac{d}{2}\times \cos\delta \theta_{xz}=\frac{d}{2}(1-\cos\delta \theta_{xz}) \end{array}$$

So,

(46) $$\delta d_{r}=d(1-\cos\delta \theta_{xz})$$

(47) $$\begin{array}{ll} \delta x=\left|\tilde{x}-x\right| & =\left|\left(h\cdot \frac{\varphi_{1,2}}{\varphi_{5,6}}\right)\cdot (1-\frac{{\tilde{d}}_{5,6}}{{\tilde{d}}_{1,2}})\right|\\ & =\left|\left(h\cdot \frac{\varphi_{1,2}}{\varphi_{5,6}}\right)\cdot (1-\frac{d+\delta d_{r}}{d})\right|\\ & =h\cdot \frac{\varphi_{1,2}}{\varphi_{5,6}}\cdot \frac{\delta d_{r}}{d} \end{array}$$

In Equation (47), $\delta d_{r}/d$ is the rotation angle error factor, where it can be seen that the rotation angle error of the USBL rotating device will produce the spacing error of the array elements, and the array element spacing error is one of the important parameters affecting the positioning accuracy of the USBL. The influence of the rotation angle error on the positioning accuracy of the USBL will be discussed by the numerical simulation method.

It can be seen from Table 5 that a 1° rotation angle error can produce a positioning error of about 0.15‰ in the horizontal distance in the rotating and array element reuse model. That is, a rotation error of 1° can cause about a 1.5 m horizontal positioning error in the horizontal positioning distance of 10,000 m. Usually, the rotation error will be less than 0.1°, and the horizontal rotation position error of 10,000 m at this level will cause a horizontal positioning error of 0.015 m, which is a very small error value. This error can be neglected even in high-precision, full-depth positioning operations. It can be seen that the rotation angle error of the USBL rotating device was less than 0.1°, which has little effect on the horizontal positioning accuracy in the USBL positioning model based on the rotary and array reusing elements method.

## 4. Simulation and Verification

In order to analyze the influence of the element spacing error δd on the positioning accuracy of the USBL, considering the wavelength error δλ, ranging error δR, and element spacing error δd of the underwater acoustical signal, this paper used the numerical simulations to set the elements spacing error of different orders of magnitude and then evaluated their impact on the USBL positioning accuracy. In addition, in order to verify the effectiveness of the proposed positioning calculation model in this paper, it was also compared with the existing USBL positioning calculation model.

**Figure 10:**
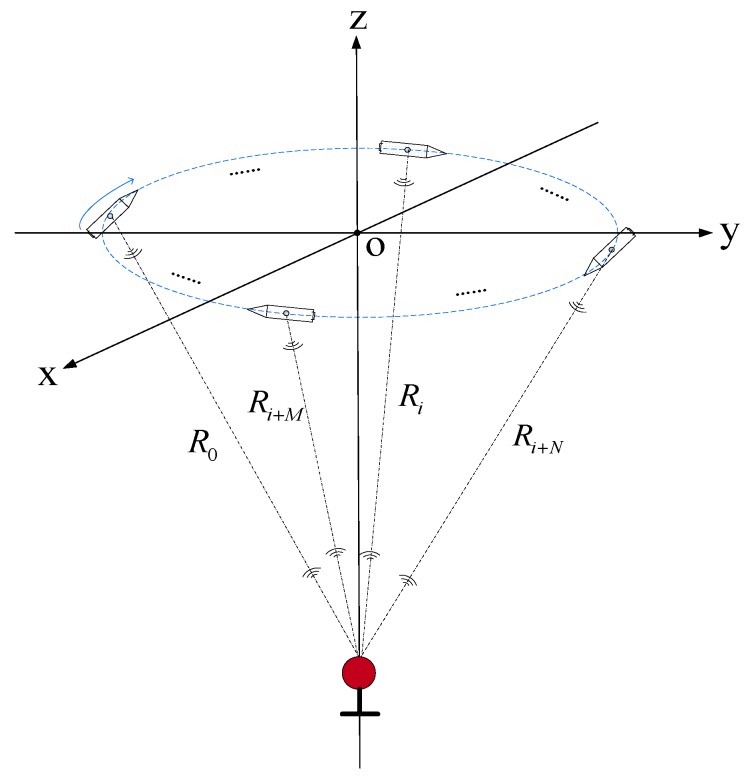
The sailing schematic diagram of the mother ship in the traditional model based on slant range and azimuth method.

Since all USBL positioning calculation methods have a slightly lower accuracy in the vertical direction than other methods, this paper only examined the positioning accuracy of the USBL positioning calculation model in the horizontal direction. The USBL solution model proposed in this paper was mainly used to eliminate the spacing error of the array elements δd and the wavelength error δλ of the underwater acoustic signal. In the simulation, the influence of the installation error angle on the positioning accuracy was not considered, that is, the installation error was assumed to be fully compensated and that there was no error in the phase difference measurement. The sailing schematic diagram of the mother ship based on the traditional model of the slant range and azimuth is shown in Figure 10.

The target was mounted with a transponder that received the underwater acoustic signal from the acoustic transmitter of the acoustic array on the mother ship, and responded to the acoustic array with an acoustic signal. In this process, the distance between the target and the USBL acoustic array was measured, and the phase difference of the acoustic array was measured when the USBL acoustic array received the underwater acoustic signal of the transponder. 

**Figure 11:**
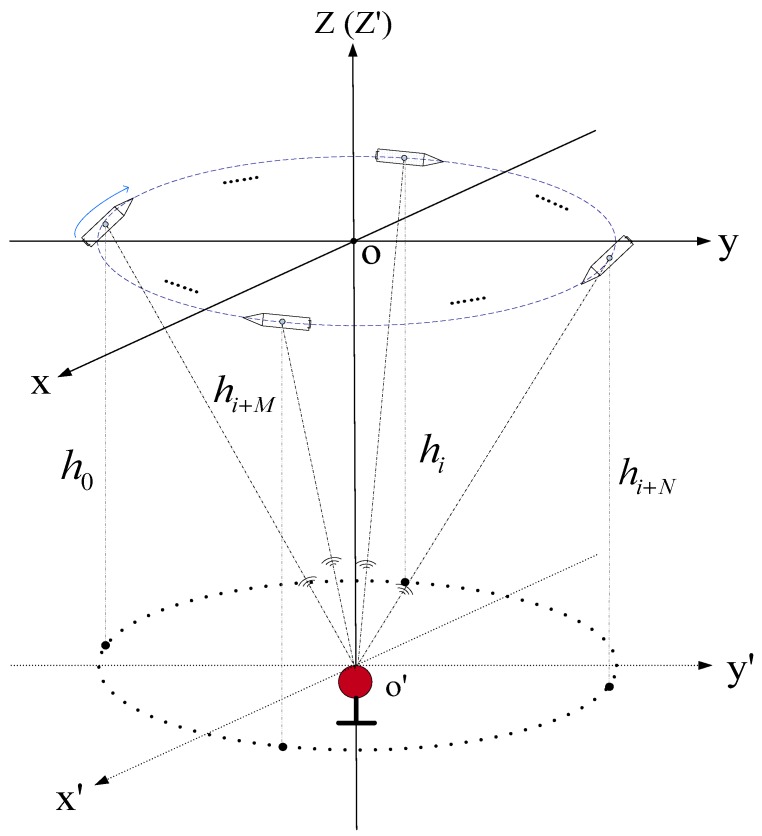
The sailing schematic diagram of the mother ship based on the rotating array and reusing elements method.

Figure 11 is the sailing schematic diagram of the mother ship based on the rotating array and reusing elements method. 

The underwater targets can adopt the beacon mode. The USBL acoustic array realizes the measurement of the phase difference by passively receiving the underwater acoustic signal from the beacon, thereby achieving an accurate measurement of the underwater target orientation. At the same time, the depth value $h_{T}$ of the underwater target is measured by a high-precision depth gauge and is sent to the USBL acoustic array through the underwater acoustic signal, and the depth value $h_{A}$ of the acoustic coordinate origin of the USBL acoustic array is recorded at the moment of the phase difference measurement (also measured by a high-precision depth meter). The distance difference $h=-\left|h_{T}-h_{A}\right|$ between the origin of the *U-*frame and the underwater beacon is considered as the z-axis coordinate value of the target in the *U-*frame.

**Figure 12:**
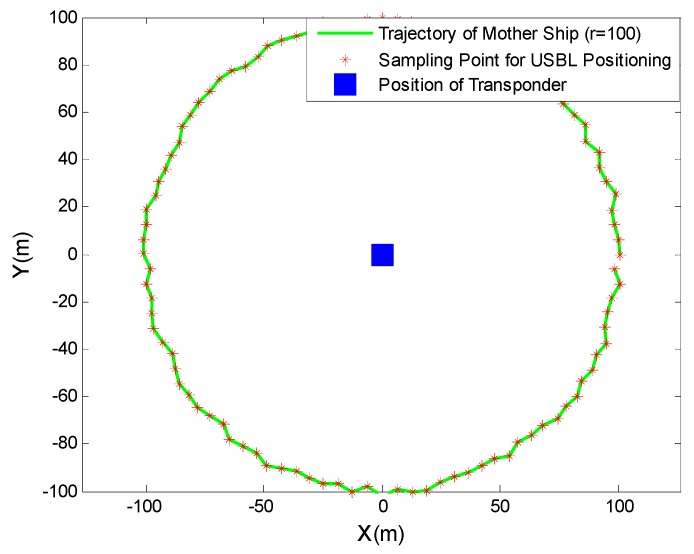
Navigational trajectory diagram of the mother ship with the circumnavigation radius of 100 m.

The simulation parameters were set as Table 6,

In the circumnavigation sailing process of the mother ship, the circumnavigation radius was designed as 100 m, 500 m, and 1000 m, respectively, and the USBL positioning accuracy under different positioning distances was investigated. Figure 12 shows the navigational trajectory diagram of the mother ship with the circumnavigation radius of 100 *m*.

### 4.1. Simulation Experiment 1

**Figure 13:**
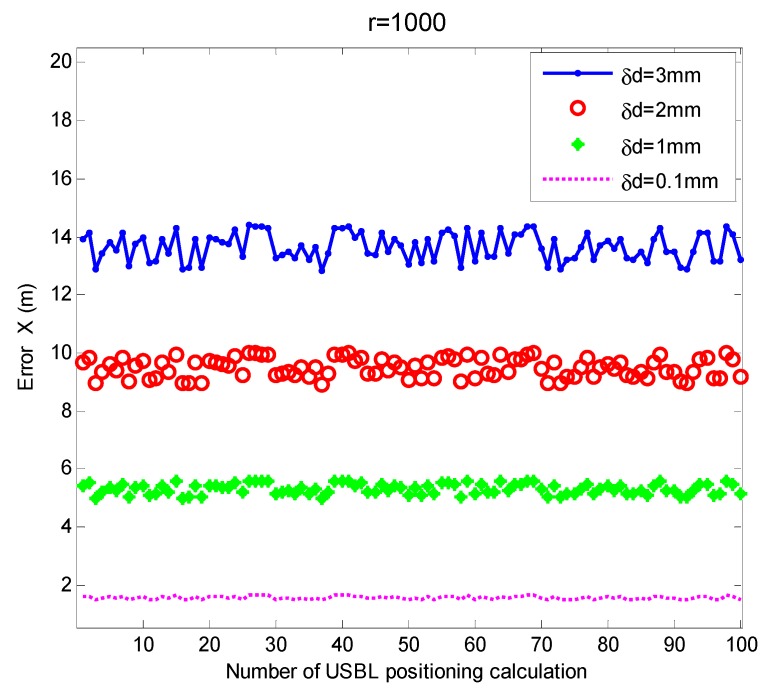
Schematic diagram of the influence of array element spacing error on positioning accuracy (based on the slant range and azimuth method).

In order to study the influence of the spacing error of the acoustic array elements of different orders of magnitude on the USBL positioning accuracy of the traditional USBL positioning model based on the slant range and azimuth method, in this simulation, the USBL acoustic array was designed with a 4-elements acoustic array structure, and the positioning calculation was based on the slant range and azimuth method. Assuming that the spacing errors of the two elements were equal, that is $\delta d=\delta d_{1,2}=\delta d_{3,4}$, and the error values were set to 3 mm, 2 mm, 1 mm, and 0.1 mm, respectively, we investigated the influence of the element spacing error of different orders of magnitude on the USBL horizontal positioning accuracy, the result show in Figure 13.

It can be seen from Figure 13 that in the case of δd, at a horizontal distance of 1000, when δd = 3 mm, the average positioning error of USBL in the x-axis direction was 13.62 m, and when it was 2 mm, the average positioning error of the USBL in the x-axis direction was 9.46 m. When δd = 1 mm, the average positioning error of the USBL in the x-axis direction was 5.30 m. When δd = 0.1 mm, the average positioning error of the USBL in the x-axis direction was 1.56 m. The mean square error was 0.43 m, 0.30 m, 0.17 m, and 0.049 m, respectively. It can be seen that the element spacing error is one of the main error sources of the USBL positioning system. The larger the element spacing error, the larger the positioning error. A 1 mm element spacing error can cause about a 6 m horizontal positioning error at the positioning distance of 1000 m. It can be seen that the positioning error is a steady state error from the theoretical analysis and simulation.

### 4.2. Simulation Experiment 2

In order to verify the effectiveness of the USBL positioning model based on the rotating array and reusing elements method proposed in this paper, we analyzed the mother ship’s horizontal positioning accuracy under different positioning distances. For this reason, the traditional positioning error based on the slant range and azimuth method and Sun’s method in the same environment were simulated and compared.

**Figure 14:**
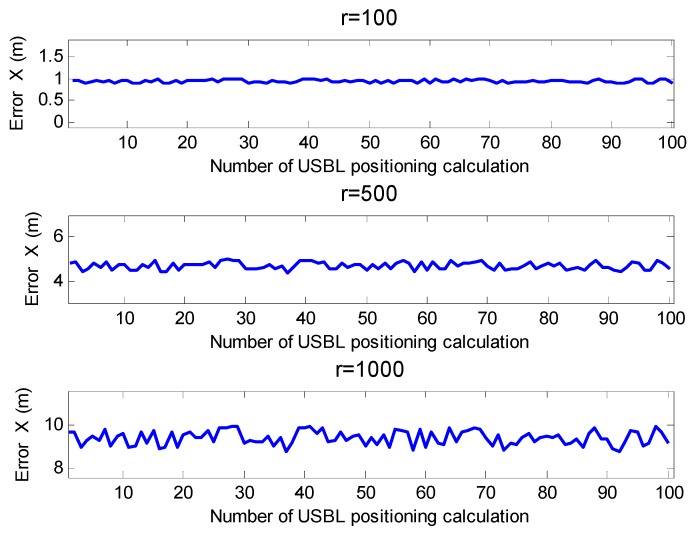
Horizontal positioning error at different positioning distances (based on the slant range and azimuth method).

Figure 14 shows the positioning error of the target in the x-axis of the *U-*frame in the case where the circumnavigation radius of the mother ship was 100 m, 500 m, and 1000 m, respectively, with the element spacing error of δd = 2 mm and it was not calibrated. It can be seen from Figure 14 that when the circumnavigation radius of the mother ship was 100 m, the positioning error of the USBL in the *U-*frame (x-direction) was about 1 m. When the circumnavigation radius of the mother ship was 500 m, the positioning error of the USBL in the USBL frame (x-direction) was about 5.2 m. When the circumnavigation radius of the mother ship was 1000 m, the positioning error of the USBL in the USBL frame (x-direction) was about 10.4 m. This positioning error is related to the distance of the target transponder, and the positioning error was about 1% of the slant distance. This is a relatively large error in a long distance and high-precision positioning environment.

**Figure 15:**
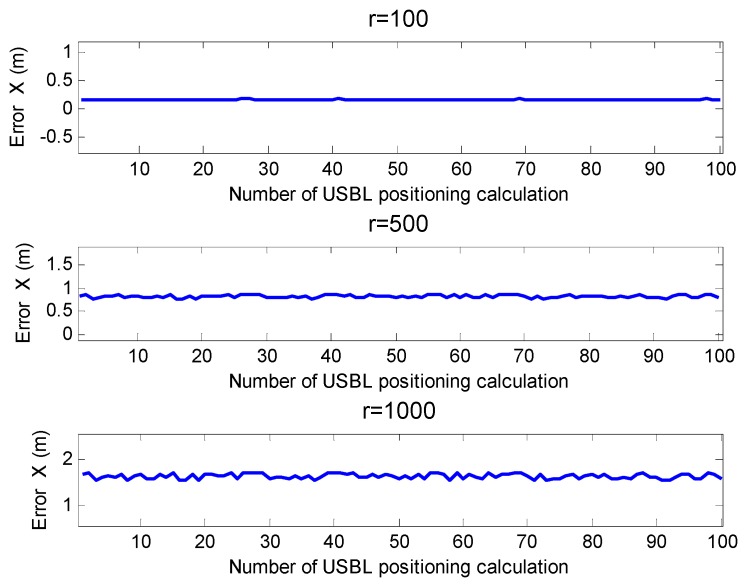
Horizontal positioning error at different positioning distances (by Sun’s method).

In [23], the geometrical element spacing of the USBL could be calibrated within 0.12 m. Figure 15 is the result of the simulation of the USBL horizontal positioning according to Sun’s method. It can be seen from Figure 15 that this method could greatly improve the positioning accuracy, especially in the case of a horizontal distance of 1000 m, where the horizontal positioning accuracy (x-axis direction) could reach 2.6 m.

**Figure 16:**
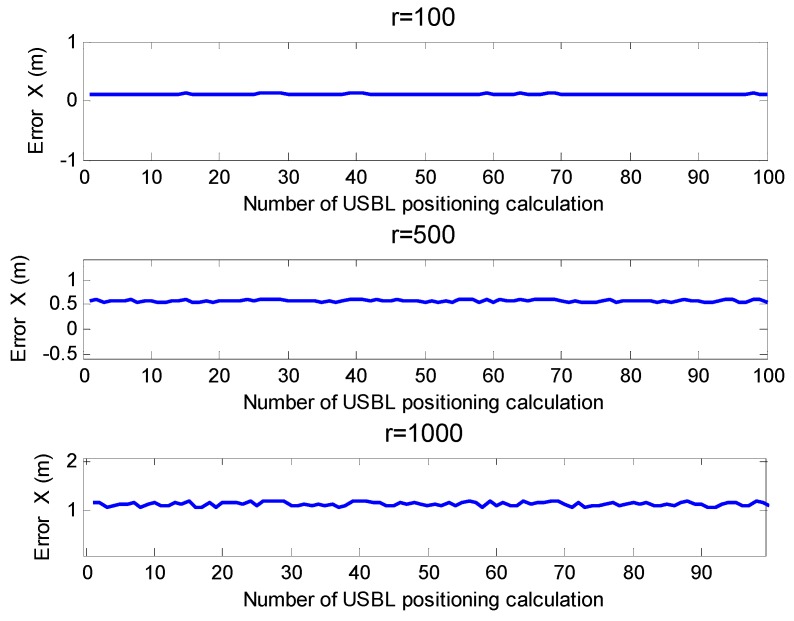
Horizontal positioning error at different positioning distances (based on the rotating array and reusing elements method).

Figure 16 is the horizontal positioning error diagram of the USBL positioning model based on the rotating array and reusing elements method proposed in this paper. It can be seen from Figure 16 that the positioning error was 0.12 m when the horizontal bypass distance was 100 m. When the horizontal bypass distance was 500 m, the positioning error was 0.57 m, and when the horizontal bypass distance was 1000 m, the positioning error was 1.14 m.

### 4.3. Simulation Experiment 3

**Figure 17:**
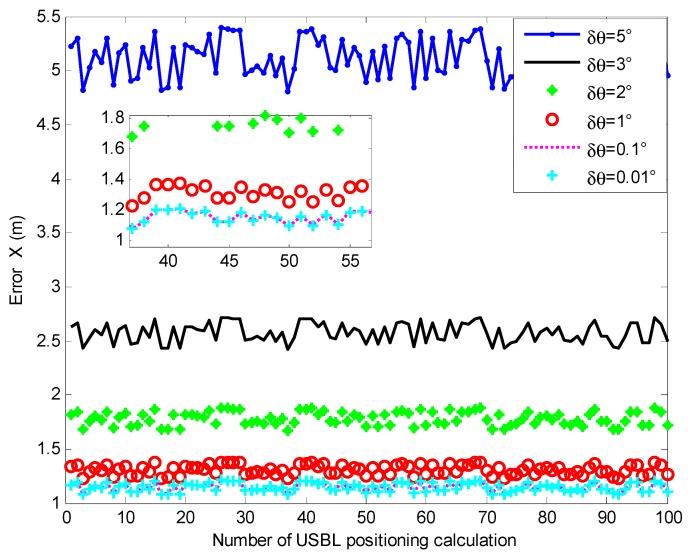
The influence of rotation angle error on USBL horizontal positioning accuracy.

In order to study the influence of the rotation angle error on the USBL positioning accuracy based on the rotating and array elements reuse method proposed in this paper, this experiment simulated the USBL rotation angle error of different magnitudes. The USBL acoustic array was designed with a 2-element structure, as shown in Figure 5, using the positioning calculation method based on depth and phase ratio information. The depth of the transponder h was 100 m, the depth measurement error δh was 0.001*h+0.01 *m*. The mother ship carrying the USBL acoustic array slowly sailed around the underwater transponder with the radius of the bypass r set as 1000 m. The rotation angle error values of the USBL rotating device were set to 5°, 3°, 2°, 1°, 0.1°, and 0.01°, respectively. We investigated the effect of the USBL rotation angle error δθ at different magnitudes on the USBL horizontal positioning accuracy. Figure 17 presents the horizontal positioning error that considers the influence of different rotation angle errors at the horizontal distance of 1000 m.

It can be seen from Figure 17 that when the USBL rotation angle error was 5°, the x-direction positioning error of the USBL based on the rotary and element reuse model was about 5.3 m. The horizontal positioning errors were 2.6 m, 1.8 m, and 1.3 m when the USBL rotation angle error was set to 3°, 2°, and 1°, respectively. When the rotation angle error was 0.1° and 0.01°, the USBL horizontal positioning error was less than 1.2 m.

## 5. Conclusions

The theoretical analysis and simulation experiments showed that the element spacing error was the main error source of the USBL positioning system. Under the condition of a positioning range of 1000 *m*, the array element spacing of 250 mm, and regardless of other error terms, a 1 mm element spacing error can cause a horizontal positioning error of about 4 m in the USBL positioning model based on the slant range and azimuth method. Additionally, there was a relationship between the error and the distance of the positioning distance where the larger the distance, the larger the positioning error. The expansion of the spacing of the array elements can suppress its influence on the USBL positioning error to a certain extent, but it cannot substantially eliminate the influence of the spacing error of the array elements. Moreover, the expansion of the spacing of the array elements will cause phase difference ambiguity, and the USBL receiving array will become huge, which will destroy the portability of the USBL. 

A new USBL positioning model based on the rotating array and reusing element method was proposed in this paper. Only two receiving array elements are required in this method. The simulation showed that for the USBL positioning model based on the method of the rotating array and reusing elements method in the range of 100 m, 500 m, and 1000 m in the USBL positioning system with a spacing of 250 mm, regardless of other errors, the positioning error can theoretically be reduced to 0.12 m, 0.57 m, and 1.14 m, respectively. In the case of considering the rotation angle error of the USBL rotating device and the depth measurement error, in the horizontal positioning environment of 1000 m, when the rotation angle error was less than 0.1°, the underwater positioning error was less than 1.2 m. The rotation angle error of the USBL rotating device was less than 0.1°, which is technically easy to implement. The model proposed in this paper can completely eliminate the influence of the wavelength error of the underwater acoustic signal and the error of the spacing of array elements on the positioning accuracy of the USBL. The positioning method has the performance of high precision in the long distance, which has important engineering application value, and provides a new idea for the engineering design of the USBL underwater positioning system.

## Figures and Tables

**Table 1:** Error parameters.

Error Term	δT (ms)	δC (m/s)	δd (mm)
Error value	1	0.05	2

**Table 2:** Location environment and equipment parameters.

C (m/s)	R (m)	d (mm)
1500	1000	250

**Table 3:** Horizontal positioning error of USBL caused by the spacing error of the array elements. (d=250 mm, x-axis direction).

d=250 mm									
x (m)	10	50	100	200	500	1000	2000	3000	4000
δx (m) δd=2 mm	0.0794	0.3968	0.7937	1.5873	3.9683	7.9365	15.8730	23.8095	31.7460
δx (m) δd=1 mm	0.0398	0.1992	0.3984	0.7968	1.9920	3.9840	7.9681	11.9522	15.9363
δx (m) δd=0.12 mm	0.0048	0.0240	0.0480	0.0960	0.2399	0.4798	0.9595	1.4393	1.9191

**Table 4:** Horizontal positioning error of the USBL caused by the spacing error of the array elements. (d=500 mm, x-axis direction).

d=500 mm									
x (m)	10	50	100	200	500	1000	2000	3000	4000
δx (m) δd=2 mm	0.0398	0.1992	0.3984	0.7968	1.9920	3.9841	7.9681	11.9522	15.9363
δx (m) δd=1 mm	0.0199	0.0998	0.1996	0.3992	0.9980	1.9960	3.9920	5.9880	7.9840
δx (m) δd=0.12 mm	0.0024	0.0120	0.0239	0.0479	0.1199	0.2399	0.4799	0.7198	0.9598

**Table 5:** Analysis of the influence of the USBL rotation angle error on the positioning error.

NO.	$\delta \theta_{xz}\;(\text{°})$	$\delta \theta_{xz}\;(\mathrm{rad})$	$\delta d_{r}\;(\mathrm{mm})$	$\delta d_{r}/d\;(‰)$
1	5	0.087266	0.951325	3.805302
2	3	0.052360	0.342616	1.370465
3	2	0.034907	0.152293	0.609173
4	1	0.017453	3.807621 × 10^−2^	0.152305
5	0.1	0.001745	3.807717 × 10^−4^	1.523087 × 10^−3^
6	0.01	0.000175	3.807718 × 10^−5^	1.523087 × 10^−5^

**Table 6:** Simulation parameters.

Parameter	Value
Depth of transponder (beacon) (m)	100 ± 2
Circumnavigate sailing radius of the mother ship (m)	100, 500, 1000
Water acoustic velocity (m/s)	1500
Water acoustic velocity error (m/s)	±0.3%v + 0.005
Time delay estimation error (s)	0.001
Depth measurement error (m)	±0.1%h + 0.01
Distance of array elements d (mm)	250
Spacing error of array elements $\delta d_{1,2}$, $\delta d_{3,4}$ (mm)	±3, ±2, ±0.1
Hydro acoustic signal frequency (kHz)	25
Hydro acoustic signal wavelength (m)	0.06
